# Multifocal Necrotizing Mayhem from Group A Strep

**DOI:** 10.51894/001c.164585

**Published:** 2026-07-31

**Authors:** Dema Boutany, Maximillian Lei, Anthony Eid, Tripti Nagar, Ammar Ghanem

**Affiliations:** 1 McLaren - Oakland

## 69

### Introduction

Necrotizing soft tissue infections (NSTIs) from group A Streptococcus (GAS) are life-threatening, with necrotizing myositis far rarer than fasciitis. Hematogenous spread from pharyngitis, particularly post-influenza, is unusual in immunocompetent adults, causing multifocal distant necrosis. Dermatologic signs such as erythema and bullae across sites offer vital early clues for prompt surgery amid high morbidity/mortality. We describe a rare case of disseminated GAS NSTI involving cervical fasciitis/myositis and remote lower extremity myositis.

### Case Description

A 30-year-old previously healthy woman presented in septic shock following influenza A and GAS tonsillopharyngitis. Symptoms included progressive odynophagia, dysphagia, hoarseness, pronounced left neck erythema, tenderness, swelling, and limited oral intake. Rapid testing confirmed influenza A and GAS. Neck CTA demonstrated diffuse inflammatory edema of superficial/deep cervical fascial planes, and chest CT showed upper mediastinal edema and small pleural effusions. Flexible laryngoscopy revealed supraglottic edema. ICU treatment involved fluids, vasopressors, and broad-spectrum antibiotics. Within 24 hours, right lower extremity erythema with hemorrhagic bullae developed with minimal tenderness and an LRINEC score of 5. Emergent exploration confirmed necrotic posterior compartment muscle confirming a diagnosis of necrotizing myositis, necessitating fasciotomies and multiple debridements. Repeat laryngoscopy identified supraglottic necrosis and neck exploration revealed purulent fascial and strap muscle necrosis. Cultures from blood, muscle, and laryngeal sites grew GAS, verifying hematogenous dissemination. Multiple debridements, supportive care, and targeted antimicrobials resulted in hemodynamic stabilization, limb preservation, extubation, and full functional recovery without recurrence.

### Conclusions

This case illustrates multifocal GAS NSTI: initial cervical erythema/swelling/fascial edema (with mediastinal extension) which rapidly progressed to hemorrhagic bullae in a remote leg, highlighting hematogenous spread from pharyngitis in an immunocompetent host. Imaging detected deep involvement before overt cutaneous changes. Despite modest LRINEC and absent classic features, this case demonstrates the necessary immediate surgical exploration with evidence of evolving bullae at any site. Dermatologic awareness of bullous/erythematous progression in septic patients, whether cervical or extremity, is essential for timely multidisciplinary intervention, preventing devastating outcomes in these fulminant infections.

